# Weight Loss in Parkinson's Disease: The Relationship with Motor Symptoms and Disease Progression

**DOI:** 10.1155/2018/9642524

**Published:** 2018-07-09

**Authors:** Maria G. Cersosimo, Gabriela B. Raina, Luis A. Pellene, Federico E. Micheli, Cristian R. Calandra, Ricardo Maiola

**Affiliations:** Parkinson's Disease and Movement Disorders Unit, Hospital de Clinicas, University of Buenos Aires, Argentina

## Abstract

**Objectives:**

To determine the prevalence of weight loss (WL) in PD patients, its relationship to the severity of motor manifestations and appetite changes.

**Methods:**

144 PD patients and 120 controls were evaluated in a single session. All subjects were asked about changes in body weight and appetite. PD patients were examined with the UPDRS-III and the Hoehn and Yahr (HY) scales. Subscores of tremor, bradykinesia /rigidity, and non-dopaminergic symptoms (NDS) were analyzed individually. Multivariable logistic regression analysis was used to determine an association between WL and PD motor manifestations.

**Results:**

48.6 % of PD patients presented WL compared to 20.8 % of controls (p < 0.001). Weight losers were significantly older and had longer disease duration, higher scores in HY stages, UPDRS-III, and NDS-subscore. Multivariable logistic regression analysis demonstrated that WL was associated with NDS-subscore (p= 0.002; OR: 1.33) and older age (p= 0.037; OR: 1.05). Appetite in PD cases losing weight was unchanged (35.7 %), decreased (31.4 %), or even increased (32.9).

**Conclusions:**

Our results showed that WL occurs in almost half of PD patients and it is largely the consequence of disease progression rather than involuntary movements or a decrease in food intake.

## 1. Introduction

Weight loss (WL) is a common clinical finding among patients with Parkinson's disease (PD) [1–4] and other neurodegenerative disorders [5, 6]. Even though the occurrence of unintended WL in PD patients has long been recognized [7–9], its underlying mechanisms are still unclear. Body weight (BW) results from the balance between energy expenditure (EE) and dietary energy intake [10]. Total EE is the amount of calories burned by an individual during one day and represents the sum of resting EE, the so-called thermic effect of food, and physical activity. Interestingly, physical activity only represents a 15 to 35 % of total daily EE whereas resting EE, which is the cost in calories necessary for the maintenance of normal body functions, accounts for 60 to 75 % of the energy used in energy-balanced individuals [10].

The question whether unintended WL in PD patients is the consequence of increased EE, decreased energy intake, or both is controversial and has yet to be elucidated [3, 11–15]. It has been suggested that WL in PD might be related to rigidity [11], dyskinesia [12], or levodopa effect [16, 17]. On the other hand, others have proposed that WL in PD patients might start years before the onset of motor symptoms [1].

In this study, we sought to determine (i) the prevalence of WL in PD patients compared to controls; (ii) the association between WL and the severity of PD motor manifestations; and (iii) its relationship to changes in appetite and food intake.

## 2. Methods

### 2.1. Subjects

We studied 144 consecutive PD patients and 120 controls. The study was approved by the ethics committee of the “Hospital de Clinicas” at the University of Buenos Aires and written informed consent was obtained from all subjects. Inclusion criteria for patients were diagnosis of idiopathic PD and a Mini Mental State score ≥ 26 [18]. Diagnosis of PD was made according to the United Kingdom Parkinson's Disease Brain Bank criteria [19]. Exclusion criteria for patients and controls included history of malignant diseases, hyper- or hypothyroidism, neurodegenerative diseases other than PD, functional surgery for PD treatment, use of neuroleptics, history of malabsorption syndrome, deglutition problems, and subjects on dietary regimens for losing or gaining weight or unable to remember their changes in BW or appetite.

### 2.2. Assessments

Patients and controls were evaluated in a single session by a neurologist specialized in movement disorders. All participants were asked what was their BW (in kilograms) at the evaluation visit (“present BW”) and 10 years earlier (“past BW”). According to the difference resulting between past and present BW subjects were divided into weight losers, weight gainers, and without changes in BW. In accordance with previous studies [20–22] we considered that there was a change in BW when a difference ≥ of 5 % between past and present BW was reported; lower differences were classified as “unchanged BW” as might not represent an actual trend towards gaining or losing weight but an occasional BW fluctuation. In addition, all subjects were asked about changes in their appetite and in the amount of daily food intake during at least the past two years; possible answers for this question were “increased, decreased or unchanged”. PD patients were also evaluated with the Hoehn and Yahr stage [23] and the Unified Parkinson Disease Rating Scale motor score (UPDRS-III) [24]. In order to determine whether BW changes were mainly associated with a certain type of PD motor manifestation, not only the total UPDRS-III score, but also the subscores of tremor (T), bradykinesia /rigidity (BR), and non-dopaminergic symptoms (NDS) were analyzed individually. T-subscore was composed of items 20 (tremor at rest) and 21 (action tremor of hands) of UPDRS-III; BR-subscore by items 22 (rigidity), 23 (finger taps), 24 (hand movements), 25 (rapid alternating movements of hands), and 26 (leg agility); and NDS-subscore by items 18 (speech), 28 (posture), 29 (gait), and 30 (postural stability). The severity of levodopa induced dyskinesias was evaluated with item 32 of UPDRS-IV [24]. Levodopa equivalent daily dose (LEDD) was calculated in all PD cases.

### 2.3. Statistical Analysis

Descriptive statistic was used to show the characteristics of the sample. Data are presented as mean ± SD or median, when appropriate. To compare the prevalence of BW and appetite changes among PD patients and controls (yes/no responses) two by two tables and the chi-square test were performed. The Mann–Whitney* U* test was used to compare quantitative variables without normal distribution. Spearman's correlation test and multivariable logistic regression analyses were performed to determine an association between WL and PD features. A p value ≤ 0.05 was considered significant. Statistical analysis was performed with the SPSS software version 20.

## 3. Results

### 3.1. Participants

Among 144 PD patients (77 males) age was 65.1± 8.5 years old (range: 38-82), disease duration 9 ± 5.9 years (range: 1-27), UPDRS-III total score 23 ± 9 (range: 2- 48), T-subscore 3.1 ± 3.2 (range: 0-23), BR-subscore 13.4 ± 5.5 (range: 2-29), NDS-subscore 3.6 ± 2.6 (range: 0- 12), and median HY stage was 2 (range: 1- 4). Ninety-one (63.2 %) PD patients exhibited wearing-off phenomena, 49 (34 %) freezing of gait (FOG), and 63 (43.8 %) presented levodopa induced dyskinesia with a median score of 2 (range= 1-3) in item 32 of UPDRS-IV. There were110 PD cases receiving levodopa (690.1 ± 265 mg/day; range 150- 1400), either as monotherapy (33 cases) or in association with dopamine agonists (DA) (61 cases), rasagiline (4 cases), DA plus rasagiline (8 cases), or DA plus entacapone (4 cases). The remaining cases were on DA (12 cases), rasagiline (7 cases), or both (6 cases).

LEDD was 768 ± 462.4 mg/day (range 100- 2390). There was a correlation between LEDD and age at disease onset (r= -0.39; p=<0.001), disease duration (r= 0.65; p <0.001), HY stage (r= 0.41; p< 00.1), subscore of non-dopaminergic symptoms (r= 0.33; p <0.001), the presence of dyskinesia (r= 0.62; p <0.001), and the occurrence of WL (r= 0.33; p <0.001).

Among the 120 controls (58 males) mean age was 62.6 ±10.9 years old (range: 37 to 90).

### 3.2. Prevalence of WL: Patients versus Controls (Figure 1)

WL was significantly higher among PD patients whereas both weight gain and unchanged BW were significantly higher in controls. We found WL ≥ of 5 % in 71 out of 144 (48.6 %) PD cases compared to 25 out of 120 (20.8 %) controls; and WL ≥ of 10 % in 43 (29.9 %) versus 9 (7.5 %) PD cases and controls, respectively. The median percentage of WL among PD patients was 11.6 % (range: 1.4-33) compared to 6.5 % (range: 2.5-14.8) in controls (p < 0.001). Gender distribution was similar among PD patients with and without WL.

### 3.3. Relationship of WL with PD Motor Features (Tables 1 and 2)

Among PD patients, weight losers were significantly older, had a longer disease duration and higher scores in HY stages, UPDRS-III, and NDS-subscore and were receiving a higher LEDD. Conversely, age at disease onset, T-subscore, BR-subscore, DK score and levodopa doses were similar in both groups (Table 1). Most PD cases that exhibited wearing-off phenomena, levodopa induced dyskinesia, and FOG and were receiving levodopa presented WL (Table 2). Among PD patients treated with DA the prevalences of WL and WG were similar to that observed in patients that were not receiving these drugs (Table 2).

There was a correlation between WL and age (r= 0.23; p = 0.005); disease duration (r= 0.28; p < 0.001); HY stage (r= 0.32; p < 0.001); UPDRS-III total score (r= 0.25; p = 0.002); NDS-subscore (r= 0.38; p < 0.001); the presence of DK (r= 0.20; p = 0.01); the fact of being on levodopa therapy (r= 0.23; p = 0.005) and LEDD (r= 0.33; p < 0.001). Conversely, no correlation was found with age at disease onset, T-subscore, BR-subscore, DK score, and levodopa doses. Multivariable logistic regression analysis showed that WL in PD patients was only associated with NDS-subscore (p= 0.002; OR: 1.33), age (p= 0.037; OR: 1.05), and LEDD (p= 0.006; OR: 1.001) adjusted by disease duration and the presence of DK.

### 3.4. Changes in Appetite and Food Intake (Table 3)

Most PD patients that presented WL reported changes in appetite (64.3 %) either increased or decreased, whereas most controls and also most PD cases without WL reported unchanged appetite (74.2 % and 71.6 %, respectively). Weight losers in the PD group reported increased, decreased, or unchanged appetite in similar proportions, while among controls there were no cases reporting WL along with increased appetite.

The prevalence of increased appetite was higher in PD cases compared to controls, but similar, in PD cases with versus without WL. Conversely, the prevalence of decreased appetite was similar in PD patients and controls, but higher in PD cases with versus without WL. Controls and PD patients without WL reported similar prevalence of appetite changes.

## 4. Discussion

The main findings of this study comprise the following: (i) WL was more prevalent and severe in PD patients compared to controls; (ii) in the PD group WL was associated with a higher NDS-subscore, older age and higher LEDD rather than tremor, rigidity, bradykinesia or dyskinesia severity; and (iii) in most PD patients WL was not the consequence of a decrease in appetite or food intake.

The present study describes the occurrence of WL in a large number of PD patients and controls and comprehensively analyzes the individual influence of tremor, bradykinesia, rigidity, and non-dopaminergic symptoms on the severity of WL.

Data from most previous studies that have addressed this topic are usually insufficient; sometimes, due to the small size of the samples studied [3, 11, 14, 25, 26], the lack of control subjects [11, 15, 16], or an undetailed description of PD motor symptoms [1, 11, 27, 28]. The prevalence and severity of WL observed in this study is consistent with that previously described in studies defining WL as a drop ≥ 5 % of BW [27]. The prevalence of WL reported in PD patients may be quite variable reaching up to 73 % depending on the criteria used for its definition [3].

We found no differences between genders in the prevalence of WL. It is not clear whether there are gender differences for WL in PD, a few studies have reported more severe WL in women [3, 8] while others failed to confirm that [26].

In line with previous studies, our results showed that the presence of DK as well as the fact of being on levodopa therapy were associated with a higher prevalence of WL [16, 17]; nevertheless, neither DK score nor daily levodopa doses were different between PD cases with and without WL. This finding suggests that WL in these cases is not related to levodopa or dyskinesia, but, perhaps, to a more advanced disease. It has been described that PD patients treated with DA may present compulsive eating leading to weight gain [29]. In this regard, we did not find a higher prevalence of weight gain among patients receiving DA; however, the presence of such compulsive behavior disorder was not specifically inquired.

Like others [3, 30], we found a higher global UPDRS motor score in PD cases with WL; however, further analysis of the subscores that compose the scale demonstrated that such difference, in our study, was at the expense of a higher NDS-subscore.

The association found between NDS-subscore and WL suggests that disease progression plays a role in the mechanisms leading to WL in PD. NDS are a group of axial motor symptoms usually resistant to dopaminergic therapy that typically mark the onset of advancing PD [31]. These clinical manifestations encompass postural instability, speech, posture, and gait disturbances and are considered the most accurate indicators of disease progression [32], whereas the appearance of NDS is in part related to disease duration, the rate at which these symptoms develop is quite variable between PD patients; therefore, their severity may be different in the setting of the same disease duration [33]. Older age was also associated with WL in both PD and control cases. It has been described that elderly subjects can experience a decrease in BW after the age of 65 years known as “unintentional WL” which has a prevalence that reaches up to 27 % in the elderly population [20, 34, 35]. The underlying mechanisms of unintentional WL are likely multifactorial including psychosocial aspects, poverty, dentition problems, depression, dementia, and the so-called “anorexia of aging” [20, 34, 36]. Older age may therefore explain WL among control subjects and probably in part accounted for WL in PD cases. Nevertheless, the higher prevalence and severity of WL observed in PD cases clearly indicates that factors other than age must be implicated in the mechanisms leading to WL in these cases. Interestingly, it has been suggested that aging plays a role in the pathogenesis of PD by interacting with the disease process at non dopaminergic structures [37, 38]. Moreover, it has been demonstrated that older age at PD onset rather than disease duration, is associated with a faster rate of disease progression, earlier appearance of NDS, cognitive impairment, and dementia [37, 39–42]. Such interplay between older age and non-dopaminergic structures compromise would result in a more severe disease; in this scenario, we found that WL in PD patients was considerably more severe. Similar to what has been proposed for WL in the setting of Huntington disease [5], WL in PD might also be considered a biomarker of disease progression resulting from increased EE caused by the disease* per se* [5, 11, 14].

Appetite among PD patients that experienced WL was unchanged, decreased, or even increased, as was observed in 32.9 % of cases. Some previous studies have already described that PD patients losing weight may present increased energy intake [3, 25, 43]. The mechanisms implicated in appetite regulation are complex and involve the interaction of a number hormones produced at different levels of the body in response to different stimuli [34, 44]. Leptin is an anorexigenic hormone secreted by adipose tissue with serum levels that are inversely correlated with body fat mass [44]. It was found that serum leptin levels in PD patients that experience WL are diminished [2, 30, 43], suggesting that the loss of body fat mass would be the primary event leading to a decrease in leptin concentration, thereby increasing appetite [2, 30, 43].

Strengths of the study are the large size of the sample, the evaluation of a control group, the assessment of BW changes in relationship to appetite, and the analysis of UPDRS-III subscores addressing their individual impact on WL. The main limitation of the study is the fact that BW and appetite changes were self-reported by participants which introduces the likelihood of inaccuracies due to poor recall; however, the inclusion of a control group may have balanced this weakness as the same drawback affected both groups.

In summary, our study shows in a large sample of patients that WL in PD is frequent and sometimes severe. The association between NDS and WL observed in this study suggests that WL is largely the consequence of disease progression rather than involuntary movements or a decrease in food intake. Further studies are necessary to address the impact of WL on the nutritional state of these patients. This may provide opportunities for medical interventions in order to diminish morbidity associated with undernutrition in patients with PD.

## Figures and Tables

**Figure 1:**
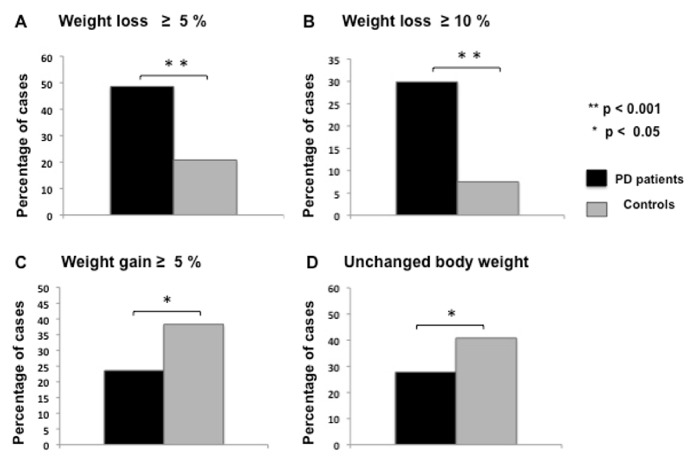
Prevalence of weight loss in Parkinson's disease (PD) patients and controls.

**Table 1:** Comparison of Parkinson's disease patients with and without weight loss.

PD patients	With WL			Without WL			p
	(n= 70)			(n= 74)			value
	Mean	±SD	Range	Mean	±SD	Range	

Age (years)	67,2	8,24	43- 81	63,23	8,47	38- 82	0.004

Age at disease onset (years)	6,39	10,64	30- 74	55,8	10,08	33- 77	ns

Disease duration (years)	10,83	5,93	1- 27	7,42	5,53	1- 24	<0.001

HY stage	2,44	0,56	2- 4	2,1	0,39	1- 3	<0.001

UPDRS –III total score	25,41	9,53	5- 48	20,84	7,93	2- 44	0.006

T-subscore	3,39	3,82	0- 23	2,89	2,68	0- 17	ns

BR-subscore	14,26	5,67	2- 28	12,62	5,43	3- 29	ns

NDS-subscore	4,73	2,9	0- 12	2,72	1,93	0-8	<0.001

DK score (item 32 UPDRS-IV)	0,99	1,01	0- 3	0,5	0,84	0- 3	ns

Levodopa dose (mg/day)	706,34	250,04	300- 1350	669,9	285,59	150- 1400	ns

LEDD	920,3	419,7	100- 2390	639,6	460,4	100- 1750	<0.001

PD= Parkinson's disease; WL= weight loss; HY= Hoehn and Yahr; UPDRS= Unified Parkinson disease rating scale; T= tremor; BR= Bradykinesia and Rigidity; NDS= non dopaminergic symptoms; DK= dyskinesias; mg= milligrams; ns= not significant; n= number of cases; LEDD= levodopa equivalent daily dose.

**Table 2:** Prevalence of weight loss among patients with motor complications and receiving dopaminergic therapies.

Motor complications / dopaminergic therapies	WL		
	Yes	No	
	n (%)	n (%)	p value
Wearing Off (n= 91)	55 (60.4)	36 (39.6)	< 0.001

DK (n= 63)	41 (65.1)	22 (34.9)	<0.001

FOG (n= 49)	34 (69.4)	15 (30.6)	< 0.001

Levodopa (n=110)	61 (55.5)	49 (44.5)	0.003

DA (n= 91)	48 (52.7)	43 (47.3)	ns

PD= Parkinson's disease; WL= weight loss; DK= dyskinesias; DA= dopamine agonists; ns= not significant; n= number of cases

**Table 3:** Changes in appetite and food intake among subjects that presented weight loss.

Appetite/ food intake	Weight losers (n= 95)		
	PD patients (n= 70)	Controls (n= 25)	
	n (%)	n (%)	p value

Increased	23 (32.9)	0 (0)	0.001

Decreased	22 (31.4)	6 (24)	ns

Unchanged	25 (35.7)	19 (76)	0.001

PD= Parkinson's disease; WL= weight loss; ns= not significant; n= number of cases

## Data Availability

The data used to support the findings of this study are available from the corresponding author upon reasonable request and the agreement of all co-authors.
